# Laminar Pipe Flow with Mixed Convection under the Influence of Magnetic Field

**DOI:** 10.3390/nano11030824

**Published:** 2021-03-23

**Authors:** Johannes Rudl, Christian Hanzelmann, Steffen Feja, Anja Meyer, Annegret Potthoff, Matthias H. Buschmann

**Affiliations:** 1 Engineering Thermodynamics, Technische Universität Bergakademie Freiberg, Akademiestraße 6, 09599 Freiberg, Germany; Johannes.Rudl@student.tu-freiberg.de; 2 Institut für Luft-und Kältetechnik Dresden, Bertolt-Brecht-Allee 20, 01309 Dresden, Germany; Christian.Hanzelman@ilkdresden.de (C.H.); Steffen.Feja@ilkdresden.de (S.F.); 3 Powder and Suspension Characterisation, Fraunhofer-Institut für Keramische Technologien und Systeme IKTS, Winterbergstraße 28, 01277 Dresden, Germany; anja.meyer@ikts.fraunhofer.de (A.M.); annegret.potthoff@ikts.fraunhofer.de (A.P.)

**Keywords:** laminar pipe flow, ferronanofluid, magnetic field, heat transfer

## Abstract

Magnetic influence on ferronanofluid flow is gaining increasing interest from not only the scientific community but also industry. The aim of this study is the examination of the potentials of magnetic forces to control heat transfer. Experiments are conducted to investigate the interaction between four different configurations of permanent magnets and laminar pipe flow with mixed convection. For that purpose a pipe flow test rig is operated with a water-magnetite ferronanofluid. The Reynolds number is varied over one order of magnitude (120–1200). To characterise this suspension, density, solid content, viscosity, thermal conductivity, and specific heat capacity are measured. It is found that, depending on the positioning of the magnet(s) and the Reynolds number, heat transfer is either increased or decreased. The experiments indicate that this is a local effect. After relaxation lengths ranging between 2 and 3.5 lengths of a magnet, all changes disappeared. The conclusion from these findings is that magnetic forces are rather a tool to control heat transfer locally than to enhance the overall heat transfer of heat exchangers or the like. Magnetically caused disturbances decay due to viscous dissipation and the flow approaches the basic state again.

## 1. Introduction

Global warming, in combination with rising worldwide energy demand, has resulted in a push for thermodynamic research to focus on developing increasingly efficient heat transfer devices. Among other options, the adding of nanoparticles to conventional fluids like water is an option for enhancing convective heat transfer. However, this strategy might be limited due to the compensation of the increase in heat conduction by viscous effects [1]. 

To follow such a path, the added nanoparticles must be ferromagnetic, which limits the choice of particle material to a few options. Among others, magnetite (Fe_3_O_4_) seems to be the most suitable raw material. A review on the subject counts 18 experimental studies on the enhancement of heat transfer in laminar and turbulent pipe flow by magnetic forces conducted between 2010 and 2020 [2]. All but one of these studies, as well as a more recent investigation [3], used water-based suspensions containing magnetite nanoparticles. Let us consider such suspensions as ferronanofluids.

It is not self-evident that the application of a magnetic field to a ferronanofluid flow causes an increase in heat transfer. It is rather the complex interaction of several forces—inertial, viscous and magnetic—as well as gravity that determines the flow situation. In addition to the Reynolds number, one of the relevant similarity numbers is the so-called magnetic number. Depending on the definition, this figure compares the magnetic force with the viscous or the inertial force.

The mentioned overview discusses three different mechanisms of how heat transfer is affected by magnetic forces. The obviously weakest mechanism is the formation of chain-like ferromagnetic nanoparticle aggregates [4]. These chains, if oriented in parallel to the heat flux, act as heat bridges. A stronger effect is the generation of secondary motions due to the viscous coupling of ferronanoparticles and the base fluid. The visualisation of such flow structures is nearly impossible due to the opacity of most ferronanofluids. Nevertheless, numerical investigations have proven their existence and their effect on heat transfer [5]. The third effect, the pinning of large amounts of ferronanoparticles to the inner wall of the pipe, occurs under the influence of comparably strong magnetic forces [4]. These dune-like structures accelerate the flow at their front and create vortex streets in their wake. Both effects change the local flow situation significantly and may induce changes of heat transfer.

Buschmann [2] points to three research gaps in the field of magnetically affected ferronanofluid flow. They are, in brief:

- investigation of the governing equations to identify the relevant parameter space,
- numerical schemes incorporating the specific mechanisms relevant to magnetically affected ferronanofluid flow, and
- experiments intended to examine the switch ability of heat transfer.

The first two points require appropriate physical experiments for validation and verification. 

The aim of this study is to investigate the influence of different magnetic fields on laminar pipe flow with mixed convection. The investigations address especially the third of the above-listed research gaps. However, its results could also support the second point by providing data for the validation of numerical results. For that purpose, experiments are carried out at Reynolds numbers covering one order of magnitude. The experimental findings are analysed with respect to the potential of magnetic forces to control heat transfer. 

## 2. Material and Methods

### 2.1. Test Rig

**Figure 1:**
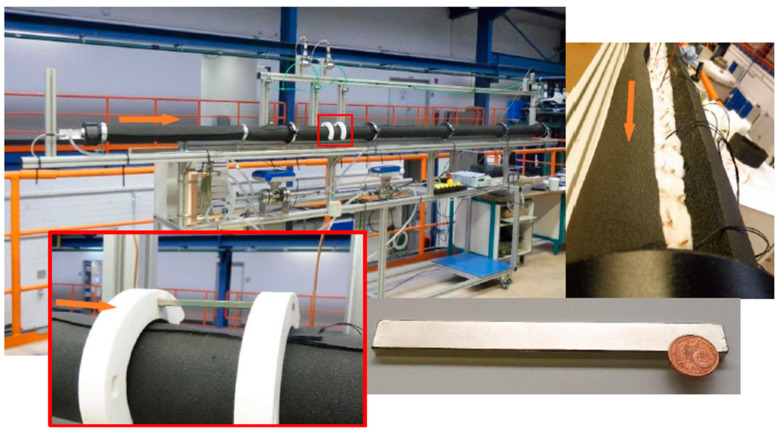
Test rig. Clockwise starting from the upper left photo: general view before closing the insulation (white: glass fibre mat, black ArmaFlex^®^ shell, wires from Pt100), NdFeB magnet, and magnet holder (single magnet above the pipe, *r_cl_* = 55 mm). Orange arrow indicates direction of flow. The blue-framed area of the magnet (lower right photo) indicates one pole area. The other pole area is opposite (the area on which the magnet lies).

Figure 1 compiles images of the test rig. The device consists of an entrance section (1000 mm), a test section (3000 mm), and an outlet section (150 mm). All parts have a circular inner cross section with a diameter of 6 mm. The wall thickness of the first two sections is 1.5 mm. The sections are thermally decoupled to avoid heat conduction between them. The length of the entrance section (PLEXIGLAS^®^) ensured a fully developed parabolic velocity profile up to the critical Reynolds number of 2300. The test section consisted of a pultruded copper pipe (Cu > 99.9%) with a roughness of *R_a_* = 0.66 µm. This extraordinarily small roughness gave at least the hope that no nanoparticles would stick to the surface due to roughness effects. This seems to be especially important in the vicinity of the magnets. The cross section of the outlet section (PLEXIGLAS^®^) is quadratic. It contains a static mixer with helical elements to create the mixing cup temperature measured at the outlet. All sections are insulated by an inner glass-fibre mat (two layers of 13 ± 2 mm, *k* = 0.04 W m^−1^ K^−1^) and an outer ArmaFlex^®^ shell (20 mm, *k* = 0.04 W m^−1^ K^−1^, armacell, Germany). A constant heat flux is generated by an electric heater coiled around the pipe. The constancy of the heat flux over the pipe mantle is ensured by the tight coiling with a very uniform pitch of about 10 mm. Moreover, the high thermal conductivity of the copper pipe (*k* = 401 W m^−^^1^ K^−^^1^) conducts the thermal energy provided by the heater nearly evenly in axial and circumferential direction. The test rig needs a filling of about 1100 mL of working fluid. 

The temperature is measured at the inlet and the outlet and additionally at 10 positions along the test section. Temperature probes (Pt100 WK 93-X, −20 °C, 150 °C, ±0.15 K, tmg GmbH, Geraberg, Germany) are placed at *x*/*d_i_* = 5.0, 18.8, 53.3, 73.3, 93.3, 115.0, 133.3, 163.3, 203.3, and 253.3 (*x* = 30 mm, 113 mm, 320 mm, 440 mm, 560 mm, 680 mm, 800 mm, 980 mm, 1220 mm, 1520 mm) on the outer mantle of the test section. The effective length of the test section employed to analyse laminar ferronanofluid flow is, at 1520 mm, longer than any other test section except one discussed in [2] and slightly longer than the one used by Tekir et al. [3].

A cooling circuit (water) provides, via a plate–heat exchanger and a thermostat (Lauda ECO RE 1050 S, temperature constancy of ±0.02 K), a constant temperature of the ferronanofluid at the entrance section inlet. The volume flux of the ferronanofluid flow is measured with a magneto-inductive flowmeter for conductive, abrasive, and aggressive liquids (KROHNE Optiflux 4100, accuracy ±0.3%). Two vibratory pumps (NME 1, 16 W, 230/240 V, 50 Hz) assembled in parallel drive the water, and an Anself pump (brushless, DC 12 V, 5 W) drive the ferronanofluid through the test circuit. Data are acquired employing a message device and the ProfiSignal software (Delphin Technology AG). The first is connected to the measurement computer, where the data are monitored in real time.

### 2.2. Magnets

Ashlar-formed permanent NdFeB magnets (HKCM^®^ Engineering e. K.) with a nominal holding force of 10.59 kg generate the magnetic field (Figure 1). Their main dimensions are 120 × 12 × 5 mm^3^ (L × B × H). The maximal operation temperature of the magnets (*t_max_* = 80 °C) limits the temperature maximum during the experiments. The magnetic flux density on the magnet surfaces amounted to 0.1215 T. The magnetic poles are axially aligned along the height (5 mm) of the magnets.

The magnets are positioned at 73.3 ≤ *x/d_i_* ≤ 93.3 (440 mm ≤ *x* ≤ 560 mm) downstream of the inlet of the test section. The poles are oriented in parallel to the test section. The distance between the centrelines of the magnets and of the test pipe *r_cl_* is 34 mm. Four magnet configurations (single magnet either above or below the pipe and two magnets above and below the pipe) for either attracting or repulsing are investigated. For the attracting and the repulsing configuration, two different poles and two equal poles, respectively, are in opposition. The main axes of the pipe and the magnets are oriented in parallel. A second distance between the centrelines of the magnets and the test pipe *r_cl_* = 55 mm is investigated for some selected cases to understand the influence of the magnetic field intensity. In all cases, the magnets are fixed in additive printed holders (Figure 1).

### 2.3. Ferronanofluid

The ferronanofluid used, MSG-W10, is a commercial product purchased from FerroTec Corporation (USA). According to the manufacturer, this aqueous magnetite (Fe_3_O_4_) suspension is stabilised by an organic chemical, anionic polymer. The appearance is an opaque, deep dark-brown. Optical inspection over a period of 6 months does not indicate sedimentation. 

The ferronanofluid is characterised by the mass concentration of magnetite nanoparticles and the fluid’s density, thermal conductivity, viscosity, and specific heat capacity. From a comparison of these results with the thermophysical properties to the data available in the literature [6], it is found that MSG-W10 is not necessarily the perfect heat transfer carrier. The reason is simply that this suspension is not made directly for convective heat transfer. However, its major advantages and why it is employed here included its high thermal stability, its availability in larger amounts, and its affordability. All three qualities recommend this suspension for more general investigations.

According to the producer, the amount of dispersed magnetite nanoparticles is between 2.8 and 3.5 vol.%. The proportion of dispersant ranges between 2 and 4 vol.%. These values are verified by exsiccating the ferronanofluid and its base fluid at room temperature and low pressure. The found mass concentrations are 22.7 and 7.6 wt.%, respectively. The difference indicates that the actual magnetite content of the ferronanofluid is about 15.1 wt.%. Considering a bulk density of magnetite between 4.9 and 5.2 kg m^−3^, the particle concentration of the ferronanofluid ranges between 2.9 and 3.1 vol.%. 

The saturation magnetisation of the ferronanofluid amounts to 186 Gs and the initial susceptibility is 0.6691 [7]. Hence, the suspension is paramagnetic. Without an additional external magnetic field, the magnetite nanoparticles are evenly distributed in space and the average magnetisation added up to zero. 

Due to the high additive content, the baseline is not water but water loaded with the same amount of stabilisers as the actual ferronanofluid. The density at 25 °C of this base fluid amounted to 1030 kg m^−3^ and that of the ferronanofluid to 1190 kg m^−3^ (Densito 30P, METTLER TOLEDO, Germany). The pH values are 8.9 and 9.1, respectively (SM Titrino 702, Metrohm AG, Switzerland). The ferronanofluid data are close to the reference values of 1.19 g/mL (22.8 °C) and a pH value of 9.0 given by the manufacturer [7].

**Figure 2:**
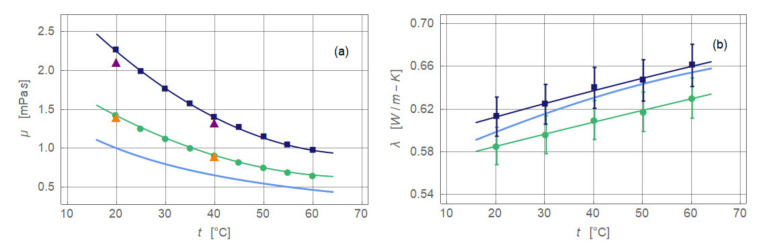

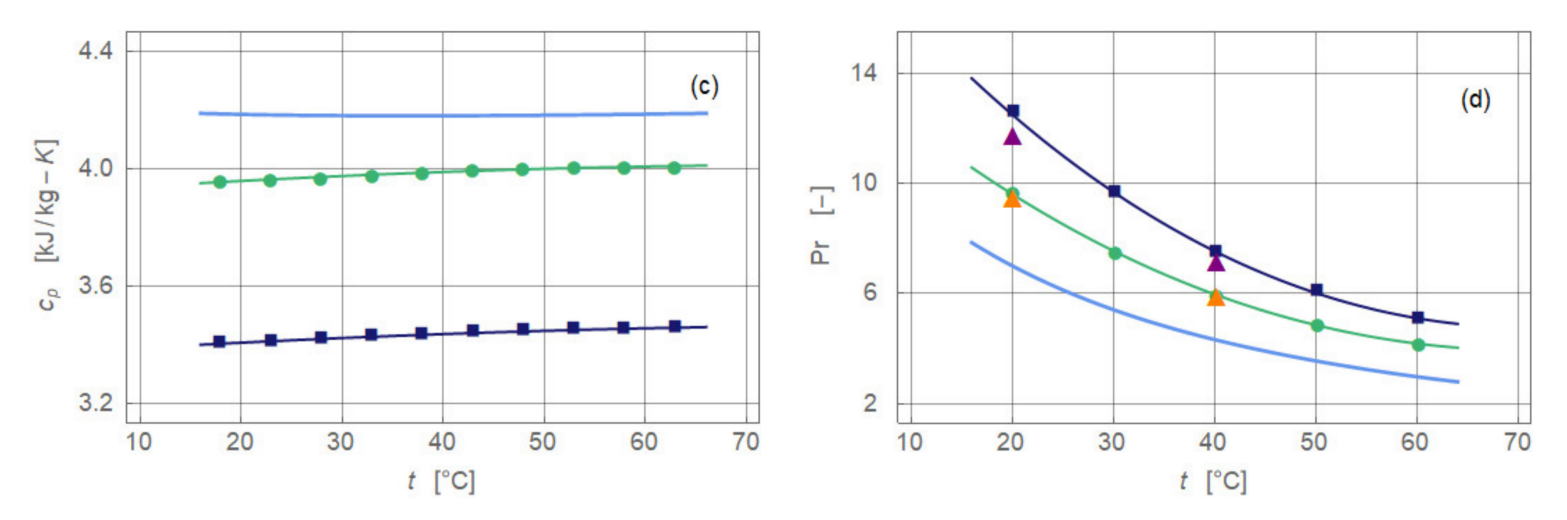
Thermophysical properties of water, base fluid and ferronanofluid. The plots show (**a**) dynamic viscosity, (**b**) thermal conductivity, (**c**) specific heat capacity, and (**d**) Prandtl number. The colours stand for DI-water (light blue, [8]), base fluid (green), and ferronanofluid (dark blue). Symbols indicate experimental data and full curves polynomial fits. Orange triangles indicate base fluid and purple triangles ferronanofluid viscosity measurements with Ubbelohde capillary.

The thermophysical properties dynamic viscosity, thermal conductivity, and specific heat capacity measured as functions of the temperature are presented in Figure 2. Each plot shows base fluid, ferronanofluid, and, for reference, the data of pure water [8]. All measurements are carried out under a zero magnetic field. The lower right plot of Figure 2 diagrams the predicted Prandtl number based on the measured data. 

Dynamic viscosity is measured with viscometer model MCR 301 (Anton Paar GmbH, Graz, Austria) and a double-gap system (DG 26.7) according to DIN 54453. Data are taken between 20 and 40 °C with an increment of 0.2 K/min and between 40 and 60 °C with an increment of 0.5 K/min at a shear rate of 50 s^−1^. The accuracy of the measurements is assumed to be less than 3% of the measurement point. The obtained data reveal that the base fluid has a mean of a 1.4-fold higher viscosity than water, while the ferronanofluid shows a 2.1-fold increase. The data found with the rheometer are confirmed by independent measurements employing an Ubbelohde capillary (metering range 1.0–10 mm^2^ s^−1^) at 20 and 40 °C. The measured data are only partially used in Figure 2. Actually, measuring points are recorded at every 0.2 K temperature difference: between 20 and 40 °C, one measuring point every minute; between 40 and 60 °C, approximately once every 20 s. Dynamic viscosity is employed in combination with the density for the determination of the Reynolds number.

Thermal conductivity is determined between 20 and 60 °C with an increment of 10 K. The measurements are carried out with a ring gap apparatus under a strategy that ensured an accuracy of 3% of the measurement value (reproducibility 1%) [9,10]. The additives already present in the base fluid lowered its thermal conductivity compared to water; temperature is kept between 2.2% (20 °C) and 4.1% (60 °C) with a mean value of 3.3%. Nevertheless, the complete suspension showed an increase in the thermal conductivity of between 0.5% (60 °C) and 2.4% (20 °C) with a mean value of 1.3%. The weak increase above water is due to the comparatively low thermal conductivity of magnetite that ranges between 6 and 7 W m^−1^ K^−1^ at room temperature [9]. The measured thermal conductivity is used to predict the local and the averaged Nusselt number.

Specific heat capacity is measured employing a differential scanning calorimeter (µDSC VII, Setaram). Data are taken between 8 and 90 °C with an increment of 5 K (0.2 K min^−1^). At each temperature level, a waiting time of 30 min ensured thermal equilibrium. Regardless, the expected error is less than 3% of the measurement value (reproducibility 1%). Adding solid particles to a liquid mostly lowers its specific heat capacity. This is also the case for the ferronanofluid investigated here. The base fluid had a specific heat capacity of about 4.7% less than that of water and about 17.9% less than that of the ferronanofluid. However, due to the higher density of the base fluid and ferronanofluid, the products (ρ c_p_)_BF_ and (ρ c_p_)_FNF_ are nearly equal, having 14 to 15% less than the equivalent values of water.

The last parameter of Figure 2 shows the Prandtl number

(1) $$\mathrm{Pr}\;=\;\frac{{\eta \;c}_{p}}{\lambda }$$

combining only thermophysical properties. It is calculated either using the measured data directly or employing the fitting functions obtained for the relevant thermophysical properties. Due to the high content of additives, the base fluid indicated an average increase of 36.2% compared to pure water. For the ferronanofluid, the average increase added up to 64.4%. Already these increases should have resulted in perceptible changes in heat transfer compared to pure water. However, such effects would not be caused by an increase of the thermal conductivity but rather by a lowering of the heat capacity and a significant increase of viscosity. As for any other nanofluid, the measurement error of the thermophysical properties generated an error of the Prandtl number which ranged around 3.3% for the base fluid and around 2.2% for the ferronanofluid. 

### 2.4. Experimental Procedure and Data Analysis

The experiments compared the ferronanofluid flow without magnetic influence with flow situations where the magnets are positioned in the mentioned four configurations. All experiments presented in the following are taken at a fixed inlet temperature of 15 °C. Hence, the inlet Prandtl number is constant throughout all experiments at *Pr_in_* = 14.2. All tests are carried out at a constant total heat input of 150 W, which is equivalent to a specific heat of 1768 W m^−2^. The ferronanofluid flow is fully laminar at the entrance of the test section in all experiments. The inlet Reynolds numbers *Re_in_* varied between 127, 237, 750, and 1234. 

All experiments are carried over one hour to ensure that fluctuations that may follow from outer influences are time averaged properly. Before each experiment, a latency of one hour is ensured with constant parameters reaching the thermal equilibrium of the test rig. The measured values of thermal conductivity and dynamic viscosity (Figure 2) fit with parabolic polynomials. Only these fits are employed to predict Reynolds, Prandtl, and Nusselt numbers.

The local Nusselt number

(2) $$Nu_{x}\;=\;\frac{h_{x}\;d_{i}}{k\left(t\right)}$$

followed from the local heat transfer coefficient *h_x_*, which is defined as

(3) $$h_{x}\;=\;\frac{q}{T_{w}\left(x\right)-\;T_{cl}\left(x\right)}$$

The local temperature of the flow on the centreline follows from the energy balance

(4) $$T_{cl}\left(x^{+}\right)\;=\;T_{in}\;+\;\left(T_{ou}-\;T_{in}\right)\frac{x}{l}$$

The temperature difference between the inner and outer wall is neglected based on the argument that the pipe material, copper, is an excellent thermal conductor and that the wall thickness, 1.5 mm, is very small. With a similar test rig the authors found that the relative error of the local Nusselt number ranged between 5.0 and 8.0% [10,11]. Therefore, the error of *Nu_x_* is set to 7.0% throughout the entire analysis. 

## 3. Experimental Results

**Figure 3:**
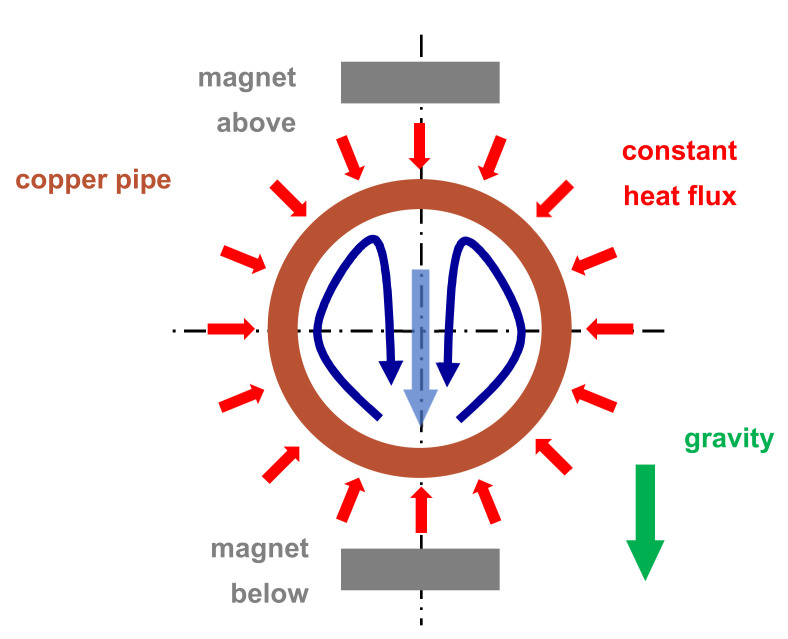
Flow situation for mixed convection (water) without magnetic influence. The main flow points into the plane of drawing. The secondary motion (counter rotating vortex pair) is shown in blue and the centrally positioned down flow by the light-blue arrow. The magnets are shown only to illustrate their position with respect to the pipe and the secondary motion. Sketch not to scale.

The experimental findings are discussed with respect to magnet configuration, intensity of magnetic field, and Reynolds number. Figure 3 sketches the flow situation for mixed convection without magnetic influence. This special form of heat transfer is explained in more detail in Section 4. The flow pattern for the different magnet configurations can only be discussed indirectly based on the revealed distributions of the local Nusselt number. 

Before giving the analysis of the experimental findings, we would like to introduce a thought experiment for the reader. Imagine you are holding between your thumb and forefinger of each hand a magnet similar to the ones used in this study. As the pole areas (blue marked in Figure 1) are brought closer together, the magnets either attract (the poles are different) or repel (the poles are the same). For the magnetite nanoparticles, the situation is different. While the magnets in the thought experiment are fixed in space, the magnetite nanoparticles which are small magnets with two poles themselves, may freely move in the suspension. Therefore, it is most likely that these nanoparticles turn under the influence of magnetic fields in a way that they will always be attracted by the magnets. The direction of force the nanoparticles experience is therefore always toward the nearest magnet.

The movement of the magnetite nanoparticles created, due to their viscous coupling with the base fluid, a magnetically driven secondary motion. Because the magnets are placed outside the pipe, this specific secondary motion is oriented toward the inner pipe wall. To maintain continuity, a counter movement away from the wall had to appear. This led to vortex systems as described by numerical simulations [5,12].

### 3.1. Magnet Configurations

**Figure 4:**
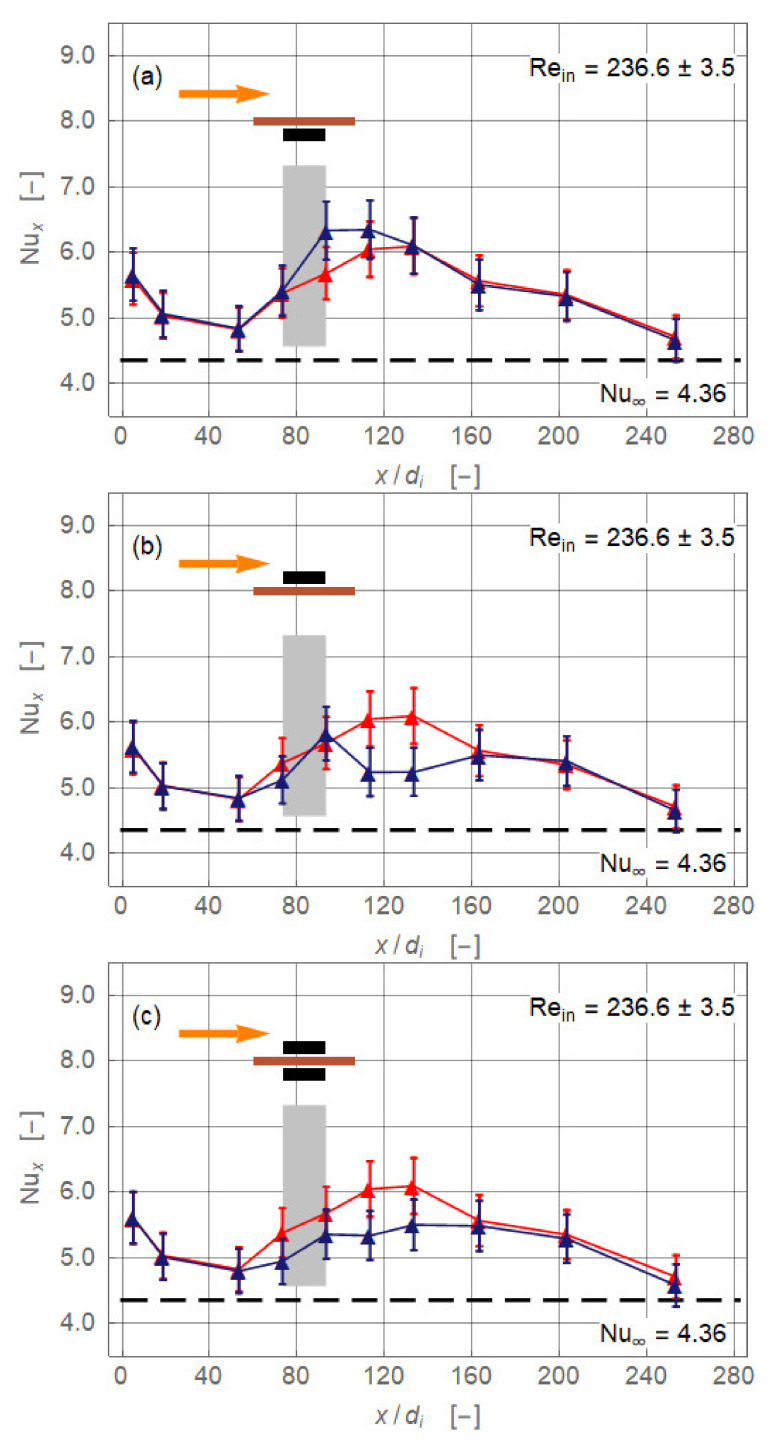

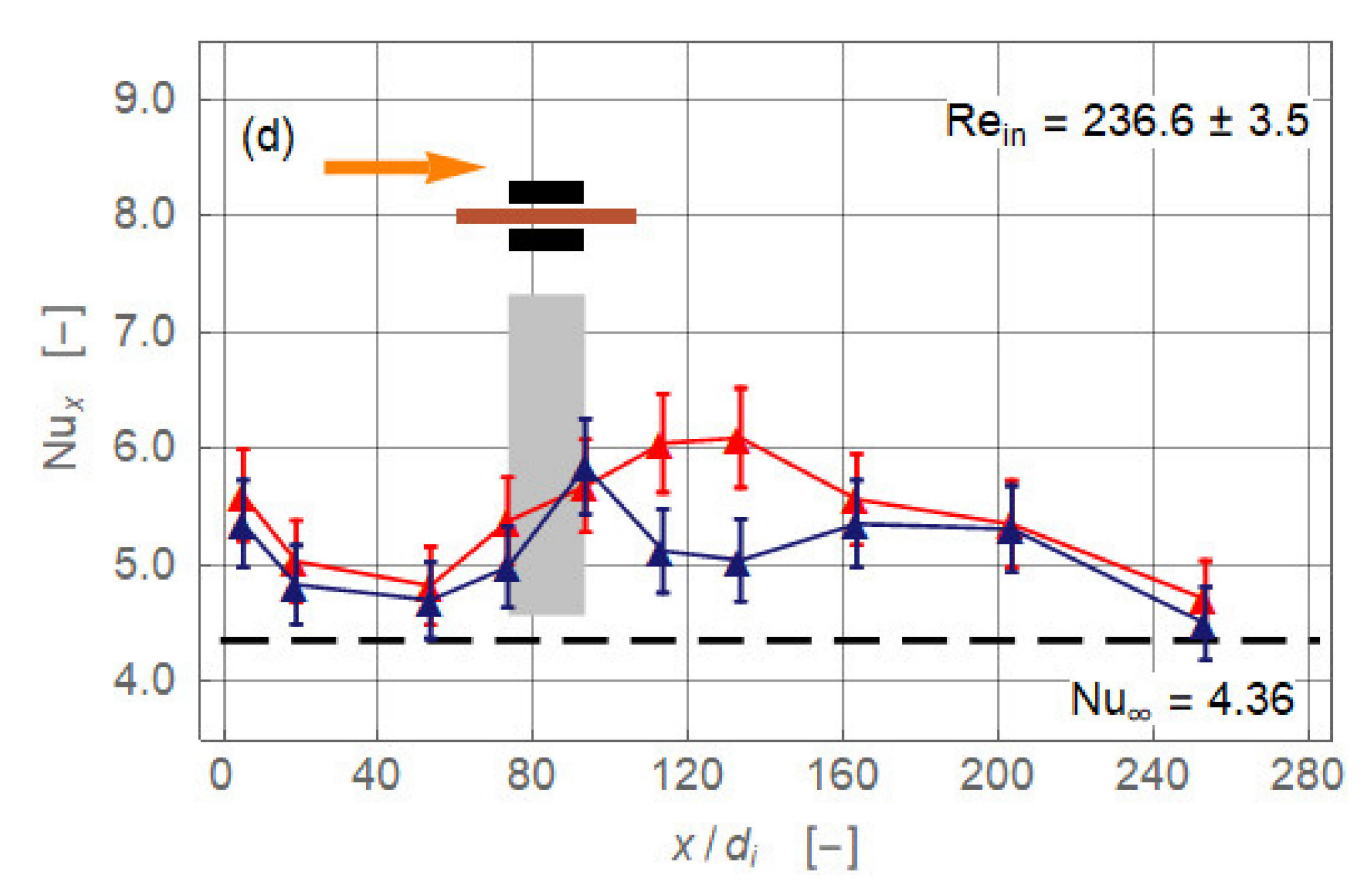
Local Nusselt number along non-dimensional axial coordinate for the magnet configurations single magnet below and above and two attracting magnets (*r_cl_* = 34 mm). The plots show (**a**) magnet below pipe, (**b**) magnet above pipe, (**c**) magnets below and above pipe attracting each other, and (**d**) magnets below and above the pipe repulsing each other. Symbols indicate experimental data. Data points connected only to improve visibility. The colours stand for ferronanofluid without magnet(s) (red) and with magnet(s) (dark blue). Orange arrow indicates direction of flow. Black rectangles show length and position of magnet(s) with respect to pipe. Grey bar indicates region of magnet(s) with respect to *x/d_i_*.

Figure 4 presents the experimentally obtained local Nusselt number for the four different magnet configurations (*r_cl_* = 34 mm, *Re_in_* = 236.6 ± 3.5). The baseline without magnetic field is identical in all plots (Figure 4, red). Up to *x/d_i_* ≅ 50, the Nusselt number baseline values are falling, approaching the limiting value $\underset{\;x/d_{i}\;\to \;\infty }{\lim}Nu_{x}\;=\;4.36$ of the exact solution for laminar pipe flow with constant heat flux [13]. The flow is entirely laminar without any secondary motion. Further downstream, the Nusselt number increased, reaching a maximum at about *x/d_i_* = 120, and decreased again. This departure from the expected monotonically falling curve throughout signal a secondary motion. The heating of the pipe wall generated a radial temperature profile that induced a pair of counter-rotating longitudinal vortices (Figure 3). This vortex pair consisted of two vertical upwardly oriented currents at the inner wall of the pipe and a stronger central down flow [13]. The laminar pipe flowed with pure thermal conduction as the heat transfer mechanism changed to a mixed convection. The thermal conduction is *supported* by a free convection. This caused an enhancement of the local heat transfer and therewith of *Nu_x_*. The more the temperature profile inside the flow is equalised, the less this augmentation is. Eventually, *Nu_x_* approached the value 4.36 again. Axial position and extent as well as the intensity of the vortex pair depended on the Reynolds number and heat input.

The uppermost diagram of Figure 4 shows the configuration where a single magnet is positioned centrally below the pipe. Magnetic and gravitational forces acted in the same direction. Therefore, the central down flow caused by the two counter-rotating gravity driven vortices is enforced by the magnet. *Nu_x_* is increased right from the upstream edge of the magnet at *x/d_i_* = 73.3. The maximal enhancement Δ*Nu_x_* = +0.65 (+ 11.5%) is achieved at *x/d_i_* = 93.3. The maximum follows a relaxation region where the Nusselt number is reduced and finally collapsed to the baseline *Nu_x_* distribution at *x/d_i_* = 113.3. Assuming that the relaxation length started at the rear end of the magnet, this length, 240 mm, is twice as long as the magnet itself. 

When the magnet is positioned above the pipe (Figure 4, second diagram), heat transfer is reduced locally. The magnetic force is now acting against gravity and the central down flow is hindered, maybe even stopped. The local Nusselt number fell below the baseline distribution. However, to establish this state takes some time and the effect is not visible before *x/d_i_* = 93.3, the rear end of the magnet. The deterioration reached, with Δ*Nu_x_* = −0.85 (−16.3%), a minimum at *x/d_i_* = 133.3 and ended at *x/d_i_* = 163.3. The relaxation length is now 420 mm, 3.5 times the magnet length, which indicated that it took quite a long time to re-establish the purely gravity-driven vortex pair. 

The last two diagrams of Figure 4 present results for the configurations with two magnets. In both cases the magnetic field should be spatially more extended than in the single magnet configurations. Therefore, its influence on the heat transfer already started at *x/d_i_* = 53.3. For the two attracting magnets the heat transfer deteriorated up to *x/d_i_* = 163.3. The minimum is reached at *x/d_i_* = 113.3 with Δ*Nu_x_* = −0.71 (−13.3%). For the repulsion case (Figure 4, last diagram), the situation is nearly identical with the configuration where the magnet is located above the pipe. The interpretation of these two cases with respect to the secondary flow is difficult. The opacity of the ferronanofluid did not allow optical access. However, one can definitively state that the gravity-driven vortex pair is significantly stalled in its motion and therewith heat transfer is lowered. Moreover, following the hypothesis from the beginning of this section—magnetite nanoparticles turn under the influence of magnetic fields such that they are always attracted by the nearest magnet—weakening of the gravity-generated secondary motion seems plausible. 

### 3.2. Intensity of Magnetic Field

**Figure 5:**
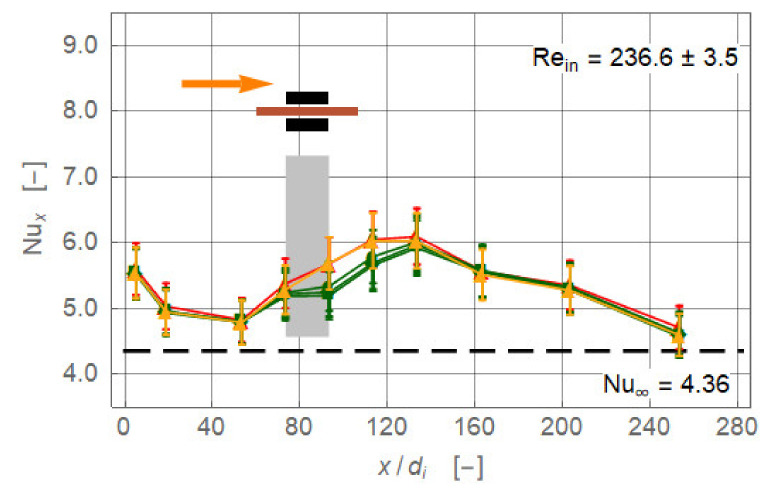
Local Nusselt number along non-dimensional axial coordinate for the magnet configurations single magnet below and above and two attracting magnets (*r_cl_* = 55 mm). Symbols indicate experimental data. Data points connected only to improve visibility. The colours stand for ferronanofluid without single magnet (red), with single magnet below (orange), and with magnet(s) (green: ■ above, ♦ above and below attracting, and ● above and below repulsing). All other symbols and marks are as in Figure 3.

Figure 5 shows results equivalent to those of Figure 4, but for *r_cl_* = 55 mm. The magnets are now approximately 20 mm further away from the flow, and their influence intuitively should already be significantly weaker. The first configuration—single magnet below the pipe—indicates actually no effect on the flow (Figure 5, orange). All other configurations cause a degeneration of the local Nusselt number, which is within experimental error and therefore identical. However, compared to the findings for *r_cl_* = 34 mm, the deterioration is weaker. Moreover, the extension of the magnetic influence ranges only from the upstream edge(s) of the magnet(s) at *x/d_i_* = 73.3 up to about *x/d_i_* = 133.3. The maximal reduction occurs in all cases at *x/d_i_* = 93.3 and ranged around *ΔNu_x_* = −0.43 (−7.5%). Further downstream, all data of the configurations with magnets collapse to the baseline results. 

**Figure 6:**
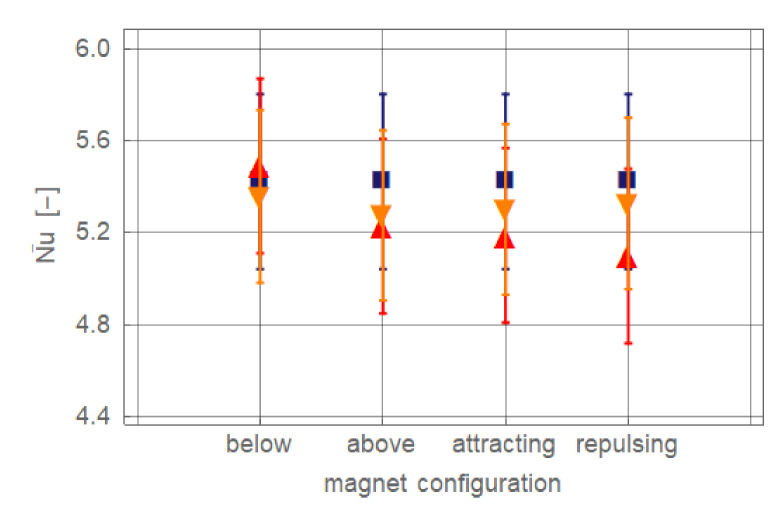
Comparison of averaged Nusselt number for different magnet configurations with *Re_in_* = 236.6 ± 3.5. Symbols indicate ferronanofluid without magnet (dark blue ■) and with magnet(s), for *r_cl_* = 34 mm (red ▲), and for *r_cl_* = 55 mm (orange ▼).

To analyse the overall effect of the magnets on heat transfer, the local Nusselt number is averaged from the first measurement position (*x*/*d_i_* = 5.0) up to the last one (*x*/*d_i_* = 253.3). For that purpose the *Nu_x_* distribution is fitted with fourth-order polynoms and integrated. The averaged Nusselt numbers for *Re_in_* = 236.6 ± 3.5 are compiled in Figure 6. Clearly the weak and nearly equal influence of the configurations with the magnet(s) distantly placed (*r_cl_* = 55 mm) is visible. No change of the averaged Nusselt number is found for the case where the magnet is positioned below the pipe with *r_cl_* = 34 mm. For all other cases, more significant effects are found than for the distant positioning. However, even for the strongest influence found—two repulsing magnets with *r_cl_* = 34 mm—the net effect with $\Delta \bar{Nu}\;=\;0.32$ (6.0%) is rather weak. 

### 3.3. Reynolds Number

**Figure 7:**
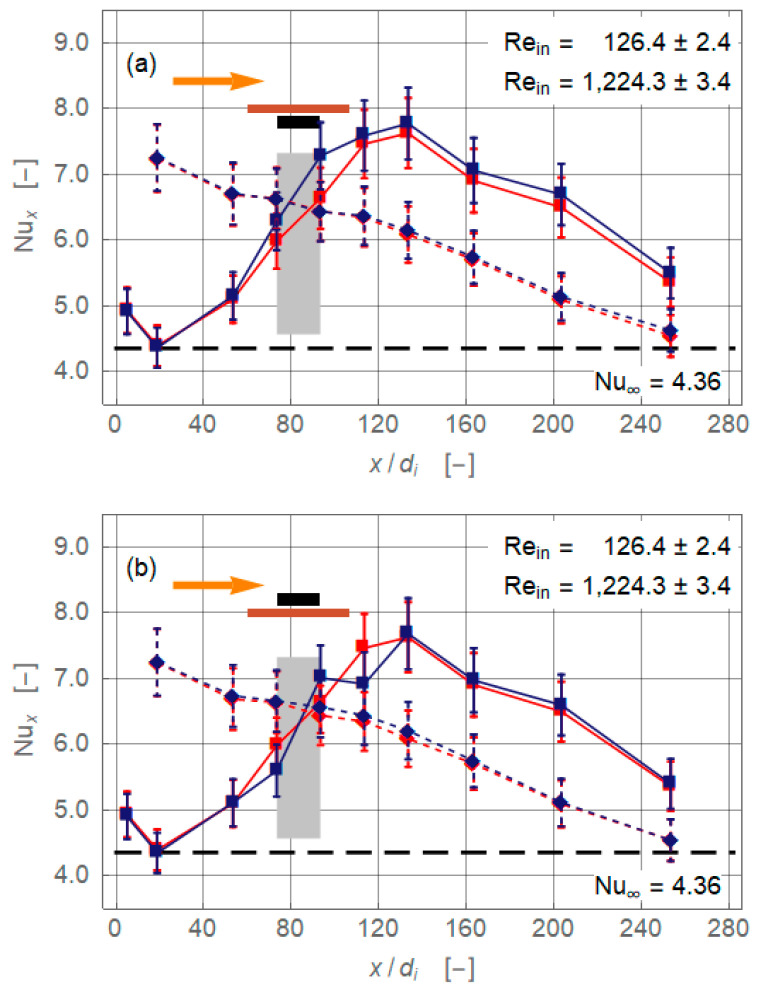

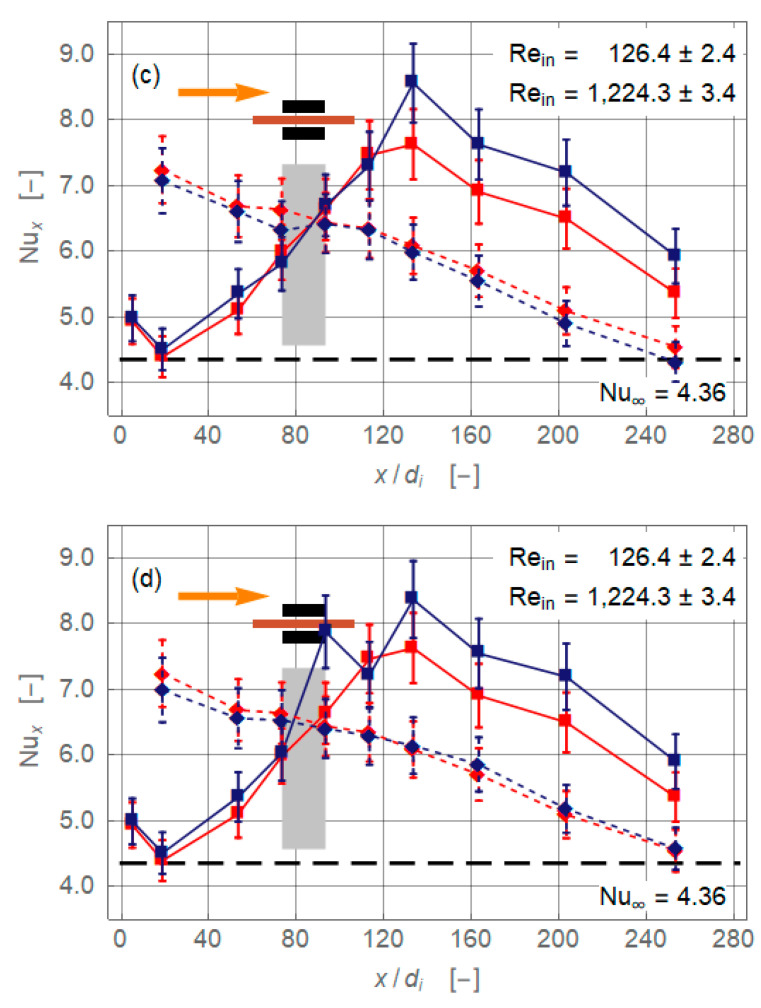
Comparison Local Nusselt number along non-dimensional axial coordinate for the magnet configurations single magnet below and above and two attracting magnets (*r_cl_* = 34 mm). The plots show (**a**) magnet below pipe, (**b**) magnet above pipe, (**c**) magnets below and above pipe attracting each other, and (**d**) magnets below and above the pipe repulsing each other. Squares indicate *Re_in_* = 126.4 ± 2.4 and lozenges *Re_in_* = 1224.4 ± 3.4.

Results at inlet Reynolds numbers of 126.4 ± 2.4 and 1224.3 ± 3.4 are compared in Figure 7. Let us first focus on the high Reynolds number data (broken lines). Contrary to *Re_in_* = 236.6 ± 3.5, the Nusselt number distributions for the different magnet(s) configurations collapse to the baseline results within the experimental error. The only exception might be the configuration with two attracting magnets (Figure 7, third diagram), where at the front of the magnets (*x/d_i_* = 73.3) heat transfer is slightly lower compared to that of the baseline. As already pointed out, the achievable changes of the heat transfer depend on the interplay of the different forces acting on the flow. Clearly, the inertial force is significantly larger than the magnetic force at *Re_in_* = 1224.3 ± 3.5, so the influence of the magnet(s) is more or less negligible. 

A comparison of the results for *Re_in_* = 236.6 ± 3.5 (Figure 4) and 126.4 ± 2.4 (Figure 7) supports the above findings. The baseline of the latter flow showed a more pronounced peak, which indicated a stronger secondary motion. Similar changes as for *Re_in_* = 236.6 ± 3.5 are found for a single magnet below the increase of *Nu_x_* in the vicinity of the magnet-and a single magnet above-deterioration downstream of the magnet. The situation is different for the configurations with two opposite magnets. In both cases, the magnets created an increase of heat transfer in a region reaching far downstream. It is open to interpretation if this effect followed from a disturbance of the gravity-driven secondary motion or if additional secondary motions are generated by the magnetic field. Samsam-Khayani et al. (2020) showed numerically the existence of such magnetically caused secondary motion/vortices in an annular horizontal tube. While these flow structures are obviously axially oriented [5], this is not necessarily the case in other flow geometries, such as rectangular channels. Again, numerically, Goharkhah & Ashjaee (2014) [14] confirmed the evolution and decay of magnetically initiated vortex systems. The flow structures these authors found for an instantaneous magnetic field are oriented normally to the main flow of a channel [14]. 

Figure 7 compares flows with Reynolds numbers differing by one order of magnitude. Both situations must be seen as laminar flows because it is rather unlikely that a fully turbulent spectrum is generated magnetically for the lower Reynolds number. Both flows are affected by secondary motions generated by a combination of gravitational and magnetic forces. In any case, these secondary motions would have decayed after a sufficient axial length and the local Nusselt number would have approached the limiting Nusselt number of 4.36. The difference between gravitational and magnetic fields and therewith their influence on the flow is that the latter is spatially confined. Any effect caused by the magnetic field will decay within a certain relaxation length following the rear end of the magnet(s). The gravitational field acts all along the pipe in a constant manner. Therefore, secondary motion induced by gravity dies out when the temperature gradient is equalised. In both cases, the viscous force played an important role in dissipating the vortex structures.

**Figure 8:**
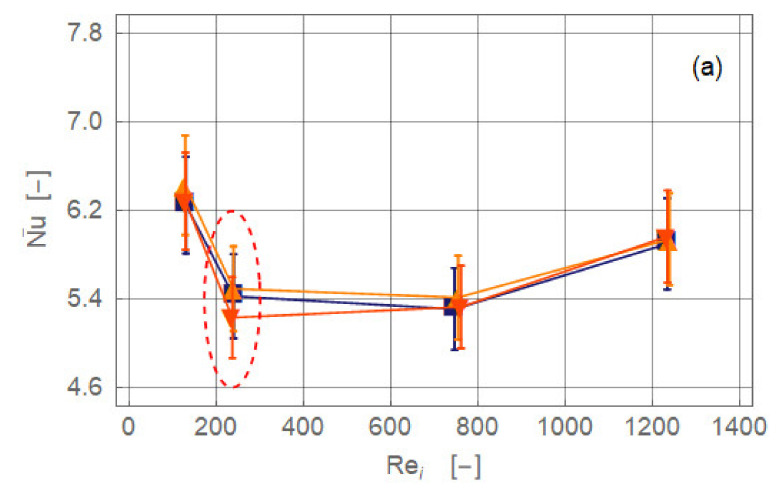

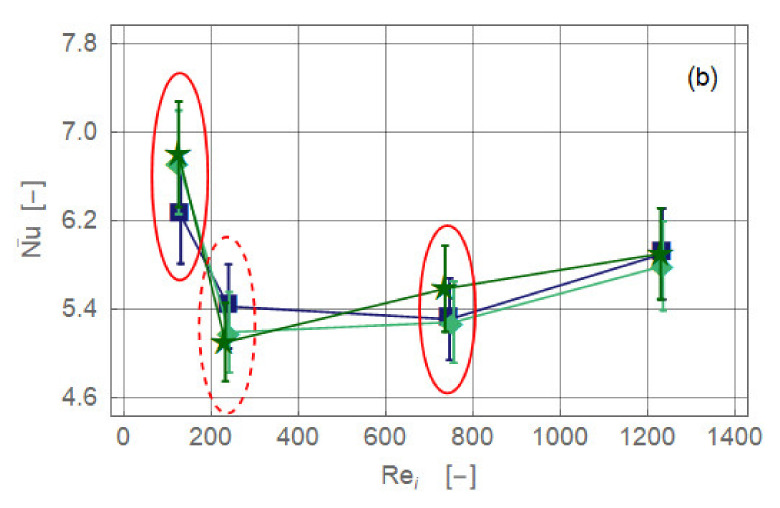
Comparison of averaged Nusselt number for different inlet Reynolds numbers with *r_cl_* = 34 mm. Symbols indicate ferronanofluid without magnet (dark blue ■, (**a**,**b**)), magnet below (orange ▲, (**a**)), magnet above (dark orange ▼, (**a**)), two magnets attracting (light green ◆, (**b**)), and two magnets repulsing (dark green ★, (**b**)). Increases are marked with full and decreases with broken ellipses.

The influence of the Reynolds number on the averaged Nusselt number is illustrated in Figure 8. As expected for a Reynolds numbers above 600, nearly no effect is visible. An exception is the configuration with two repulsing magnets (lower plot, green star at *Re_in_* = 749.9 ± 9.8). The obtained increase, with $\Delta \bar{Nu}$ = 0.28 (5.2%), is rather weak. More pronounced is the increase at the lowest inlet Reynolds number (*Re_in_* = 126.4 ± 2.4) for the attracting ($\Delta \bar{Nu}$ = 0.48, 7.7%) and for the repulsing configuration ($\Delta \bar{Nu}$ = 0.56, 8.9%). Similar minor decreases appeared only at *Re_in_* = 236.6 ± 3.5, but for the configuration where a single magnet is placed above the pipe. In summary, the higher the Reynolds number at the inlet, the lower the influence of the magnet(s) on the overall heat transfer. At low Reynolds numbers, there is little effect either positive or negative. 

## 4. Summary and Conclusions

The heat transfer in laminar pipe flow with mixed convection under the influence of magnetic fields is investigated experimentally. Local Nusselt number distributions for four different magnet configurations with Reynolds numbers stretching over one order of magnitude from about 120 to 1200 are considered. While most investigations (e.g., [2,3,15]) aim to enhance heat transfer, our study seeks to lay the foundations for controlling and switching heat transfer.

The novelty of the study is that, among others, not only laminar straight pipe flow but also mixed convection is investigated. Mixed convection is characterised by an axial and radial flow component. The effective heat transfer is a combination of conduction and convection. The first component is dominated by the thermophysical properties of the ferronanofluid, mainly thermal conductivity. The convective share follows from radially and circumferentially oriented secondary motion. It is, to the best of our knowledge, the first time that the interaction between such a secondary motion and magnetic forces is experimentally investigated. 

All magnet configurations exert a local disturbance on the ferronanofluid flow. The heat transfer is thus directly influenced in the vicinity of the magnet(s). However, the effect on the flow and therewith on the heat transfer is also nonlocal. Due to a memory effect, which is similarly known from the decay of grid turbulence [16], information is retained by the vortex structures of the secondary motion and convected downstream. Only when viscous dissipation has finished its work and destroyed this information the flow has relaxed. Downstream from this point, there should no longer be any effect on heat transfer. This nonlocal effect is strong and manifested itself at a relaxation distance of several lengths from the magnets. Following such an argument, one must keep in mind that the flow investigated here is always highly likely to be laminar. The viscous decay of laminar secondary motions, whether they originate from gravitational or magnetic forces or a combination of both, is essentially different from the turbulence cascade proposed by classical theory. 

The major experimental findings of the study are as follows:

- The pipe flow of the working fluid, a suspension of magnetite nanoparticles, exhibits a significant gravity-driven secondary motion already in the absence of magnetic influence. This motion is either hindered or assisted by the magnetic field. Which of these two cases occur and with what intensity depends on the spatial orientation of the magnetic force with respect to gravity and on the Reynolds number.
- The alteration may either be positive (enhancement) or negative (deterioration). The effect of the overall heat transfer of the pipe flow is rather weak and rarely exceeds 5% (positive or negative). The further the radial distance of the magnets from the flow or the higher the Reynolds number, the weaker are the effects on heat transfer.
- Based on data analysis, it is argued that of the three possible heat transfer enhancing mechanisms in laminar pipe flow under a radial magnetic field—viscosity-controlled percolation effect, inertia-controlled formation of secondary motion, and magnetic force-controlled dune formation—the second seemed the most likely to occur in our experiments. The first option is ruled out because of the comparably high Reynolds numbers, and the third because of the comparably weak magnetic forces.

**Figure 9:**
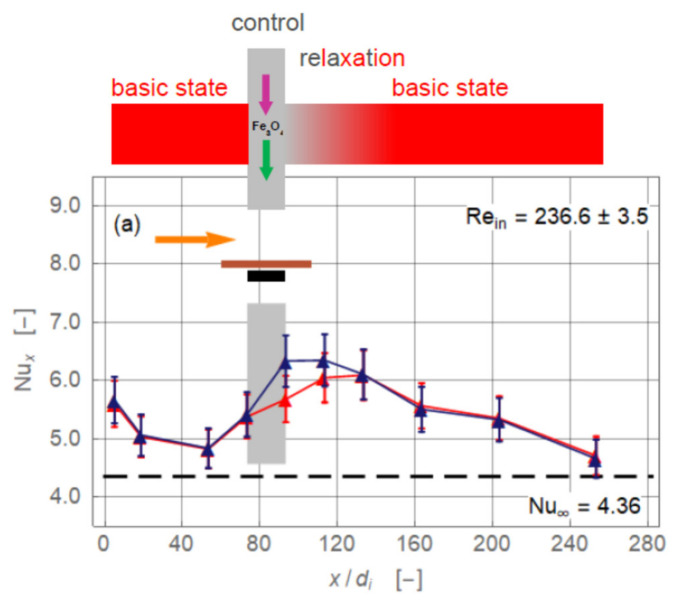

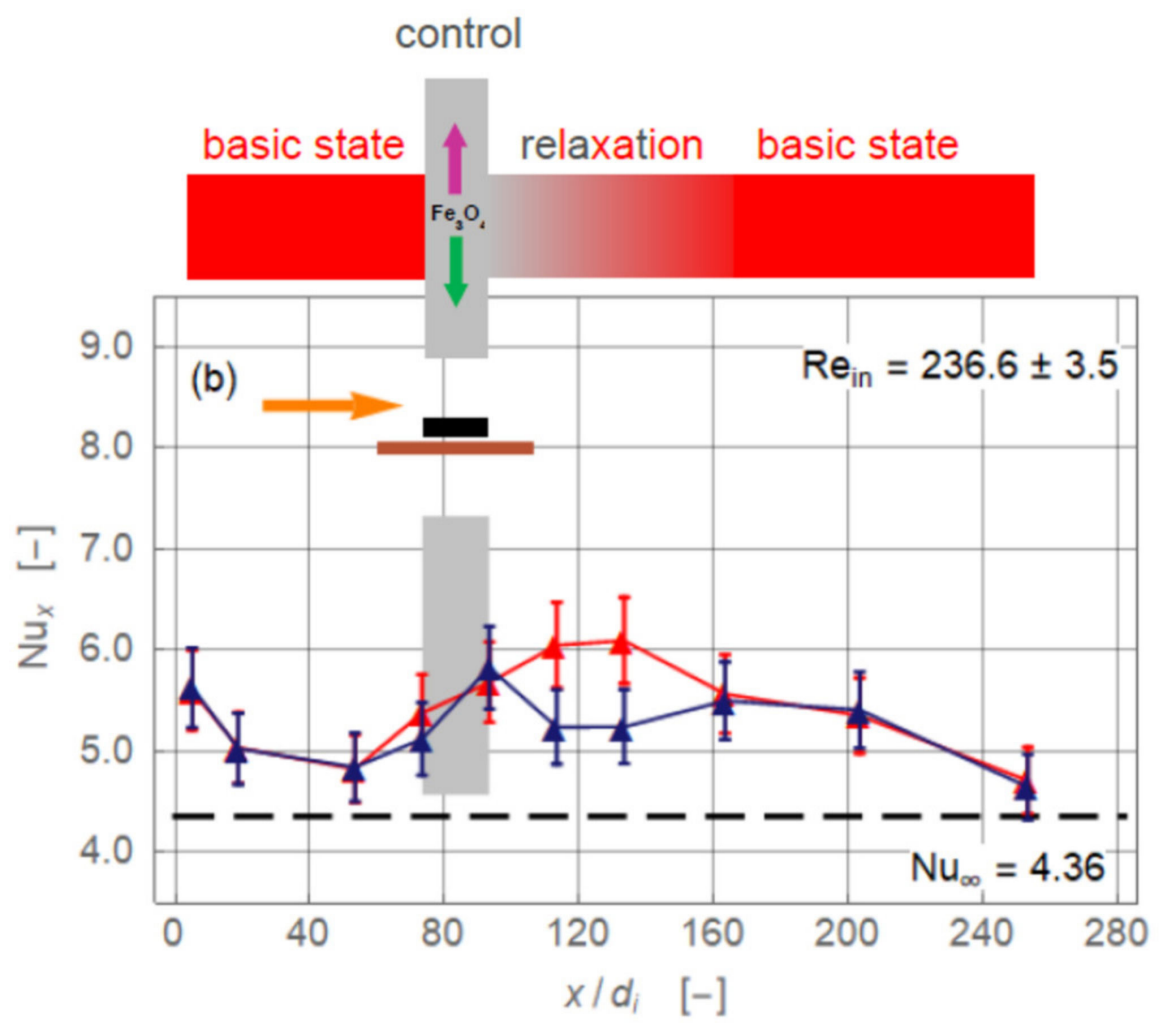
Characteristic of flow control. Magnet below pipe (**a**) and magnet above pipe (**b**). Symbols as in Figure 4. Green arrow acting on the Fe_3_O_4_ nanoparticle represents gravity and purple arrow, magnetic force. The red areas above the plots indicate the zones of the base state. Grey areas show regions where the magnets affect the flow and therewith the local heat transfer. The grey marked relaxation regions stretch well downstream from the rear ends of the magnets.

The conclusion from these findings is that magnetic forces may be seen as a tool to control local heat transfer rather than to enhance the overall heat transfer of a heat exchanger or the like. Figure 9 sketches the situation for *Re_in_* = 236.6 ± 3.5 once again. The drawings on the top of the diagrams illustrate the basic state related to the region upstream of the magnets that is disturbed by the magnetic field. Such a perturbation can lead either to an enhancement or a reduction of heat transfer. Therefore, it can be imposed with the intension *of controlling* the flow and, therewith, the heat transfer. Downstream of the magnets the disturbance decays due to viscous effects and the flow approaches the basic state again. The possibility of performing such a control with permanent magnets seems rather limited. Further research should therefore focus on the use of instantaneous magnetic fields generated by electromagnets.

## Data Availability

Data are available from MHB upon reasonable request.

## Nomenclature

*c_p_*	specific heat capacity	(J kg^−1^ K^−1^)
*d_i_*	inner diameter of test pipe	(mm)
*h*	heat transfer coefficient	(W m^−2^ K^−1^)
*K*	thermal conductivity	(W m^−1^ K^−1^)
*L*	overall length of test section	(mm)
*Nu*	Nusselt number	(-)
*Pr*	Prandtl number	(-)
*Q*	specific heat	(W m^−2^)
*r_cl_*	distance between centrelines of magnet(s) and pipe	(mm)
*Re*	Reynolds number	(-)
*t, T*	temperature	(°C, K)
*x*	streamwise coordinate	(mm)
*η*	dynamic viscosity	(kg m^−1^ s^−1^)
*ρ*	density	(kg m^−3^)
**Subscripts**		
cl	centreline	
in	inlet of test section	
ou	outlet of test section	
x	local coordinate in axial direction of pipe	
w	wall	
**Abbreviations**		
BF	base fluid	
FNF	ferronanofluid	
